# A prospective Phase II study to examine the relationship between quality of life and adverse events of first‐line chemotherapy plus cetuximab in patients with KRAS wild‐type unresectable metastatic colorectal cancer: QUACK trial

**DOI:** 10.1002/cam4.1623

**Published:** 2018-07-26

**Authors:** Shigeyoshi Iwamoto, Akira Ooki, Satoshi Morita, Hiroki Hara, Hiroaki Tanioka, Hironaga Satake, Masato Kataoka, Masahito Kotaka, Yoshinori Kagawa, Masato Nakamura, Tatsushi Shingai, Masashi Ishikawa, Yasuhiro Miyake, Takeshi Sudo, Yojiro Hashiguchi, Taichi Yabuno, Junichi Sakamoto, Akihito Tsuji, Masahiko Ando, Kensei Yamaguchi

**Affiliations:** ^1^ Cancer Center Aichi Medical University Nagakute Japan; ^2^ Department of Gastroenterology Saitama Cancer Center Saitama Japan; ^3^ Department of Gastroenterological Chemotherapy Cancer Institute Hospital of Japanese Foundation for Cancer Research Tokyo Japan; ^4^ Department of Biomedical Statistics and Bioinformatics Kyoto University Kyoto Japan; ^5^ Department of Medical Oncology Okayama Rosai Hospital Okayama Japan; ^6^ Department of Medical Oncology Kobe City Medical Center General Hospital Kobe Japan; ^7^ Department of Surgery National Hospital Organization Nagoya Medical Center Nagoya Japan; ^8^ Gastrointestinal Cancer Center Sano Hospital Kobe Japan; ^9^ Department of Surgery Kansai Rosai Hospital Amagasaki Japan; ^10^ Comprehensive Cancer Center Aizawa Hospital Matsumoto Japan; ^11^ Department of Surgery Osaka Saiseikai Senri Hospital Suita Japan; ^12^ Department of Surgery Shikoku Central Hospital Shikokuchuo Japan; ^13^ Department of Surgery Osaka Minato Central Hospital Osaka Japan; ^14^ Department of Surgery Yamagata Prefectural Central Hospital Yamagata Japan; ^15^ Department of Surgery Teikyo University School of Medicine Tokyo Japan; ^16^ Department of Surgery Yokohama Municipal Citizen's Hospital Yokohama Japan; ^17^ Tokai Central Hospital Kakamigahara Japan; ^18^ Department of Medical Oncology Kagawa University Takamatsu Japan; ^19^ Center for Advanced Medicine and Clinical Research Nagoya University Nagoya Japan

**Keywords:** adverse event, Cetuximab, chemotherapy, colorectal cancer, Quality of Life

## Abstract

A prospective trial has not been performed to investigate associations between quality of life (QOL), adverse events (AEs), and overall survival (OS) in the first‐line treatment with cetuximab plus standard chemotherapy for advanced/metastatic colorectal cancer (mCRC). Associations between patient outcome and health‐related QOL (HRQOL) together with skin toxicity‐related QOL were prospectively evaluated using EORTC QLQ‐C30 and DLQI questionnaires. One hundred and forty mCRC patients were analyzed in this study, and 87.8% received pre‐emptive skin treatment. Skin toxicity had no clinical impact on HRQOL or skin‐related QOL during the first 8 weeks and throughout the study period. An early skin reaction with a grade ≥2 at 8 weeks was significantly associated with a favorable OS compared with a grade of ≤1 (HR, 0.50; 95% CI, 0.24‐0.95; *P* = .035) and was confirmed to be an independent predictor of OS (HR, 0.48; 95% CI, 0.21‐0.97; *P *= .040). Patients symptomatic at baseline who responded to treatment had improved HRQOL compared to nonresponding patients. Severe mucositis/stomatitis had a statistically significant and clinically meaningful negative impact on HRQOL (mean changes from baseline throughout the study period in global health status were −12.64 for a grade of ≥2 vs −0.35 for a grade of 0 or 1 (*P* = .005)). In conclusion, severe early skin reactions predict favorable OS for patients treated with cetuximab plus chemotherapy without impairing QOL. In addition, mucositis/stomatitis was the most substantial AE compromising both QOL and treatment compliance.

## 1 INTRODUCTION

The standard first‐line treatment for mCRC comprises doublet chemotherapy (irinotecan‐ or oxaliplatin‐based) combined with an anti‐epidermal growth factor receptor antibody (anti‐EGFR ab: cetuximab, panitumumab) or antivascular endothelial growth factor antibody (bevacizumab), resulting in a marked improvement in the prognosis of these patients.^1^ However, complete cure from mCRC has essentially been impossible, and the main goals of various treatments are the prolongation of survival, prevention of tumor progression, improvement of tumor‐related symptoms, and maintenance of quality of life (QOL).^2^

Cetuximab has been used widely in the treatment of mCRC, and meta‐analyses supported the potential benefit of first‐line use of anti‐EGFR ab plus chemotherapy compared with bevacizumab plus chemotherapy for OS time.^3^, ^4^ In this regard, the addition of cetuximab to a standard chemotherapy regimen for mCRC has become one of the most promising regimen strategies in the first‐line treatment for mCRC patients with wild‐type RAS genotypes. Although there is undoubtedly a treatment benefit derived from cetuximab plus chemotherapy, this regimen might inevitably accompany potential AEs resulting in negative impact on QOL.

Information about the effect of treatment on health‐related quality of life (HRQOL) has recently become increasingly crucial, as patients tend to ask for more information on their QOL in conjunction with prognosis.^5^ In this regard, patient‐reported outcomes are a useful way to evaluate additional advantages and disadvantages of the treatments.^6^ Skin toxicity reactions are one of the most common anti‐EGFR ab‐related adverse events (AE)^7^ and may not only impose a negative impact on skin‐related QOL,^8^ but also may impair general HRQOL, resulting in psychological distress and avoidance of social contacts.^9^ On the contrary, other investigators have reported no statistically significant or clinically meaningful differences in terms of HRQOL between groups of patients treated with first‐line anti‐EGFR ab plus chemotherapy and chemotherapy alone.^10^, ^11^, ^12^ Additionally, there are reports that skin toxicity reactions from anti‐EGFR ab treatment were able to predict positive treatment benefits in terms of prognosis of the patients treated with those anti‐EGFR ab containing regimens.^13^, ^14^ Those findings, however, are limited by their “ad hoc”, descriptive, exploratory, and retrospective nature. To date, there have been few prospective assessments investigating HRQOL and skin toxicity.^15^ In addition, the previous analyses that have evaluated skin toxicity have not examined the association between HRQOL and all other AEs, including severe symptoms mostly caused by the concomitant chemotherapies.^15^, ^16^, ^17^, ^18^, ^19^ In this respect, a comprehensive examination of the adverse effect on HRQOL is needed to clarify the proper use of anti‐EGFR ab together with the standard chemotherapy for mCRC patients with RAS wild‐type tumors.

In this study, we have conducted a single‐arm Phase II trial, prospectively evaluating the association of survival outcome, HRQOL, and subjective and objective skin toxicity in those patients treated with cetuximab plus chemotherapy. We aim to facilitate personalized decision‐making which includes the patient's perspective and to achieve the proper management of those mCRC patients in clinical practice.

## 2 PATIENTS AND METHODS

### 2.1 Study design and treatments

The QUACK study is a multicenter, prospective, Phase II study conducted in Japan. Detailed information with respect to the study design, patient eligibility criteria, etc. has been previously described.^20^ Registered patients were treated with FOLFIRI plus cetuximab or mFOLFOX6 plus cetuximab by the physicians’ discretion in each institution according to their standard clinical practice for treating mCRC. This study has been conducted in accordance with the Declaration of Helsinki and the Ethics Guidelines for Clinical Research by the Ministry of Health, Labor, and Welfare in Japan. Informed consent was obtained from all patients before registration. The study protocol was approved by the institutional review board or ethics committee of each participating institution, and it was registered with the University Hospital Medical Information Network (UMIN) Clinical Trial Registry (UMIN000010985) on 19 July 2013.

### 2.2 Endpoints and assessments

The endpoints are the following associations: AEs and QOL, treatment efficacy and skin toxicity, and efficacy and QOL. Disease progression and the occurrence of new diseases were monitored by radiological methods (computed tomography or magnetic resonance imaging) at prechemotherapy (baseline) and every 8 weeks during the treatment period. Treatment response was evaluated by the investigator at each institution using the Response Evaluation Criteria in Solid Tumors (RECIST) version.1.1.

AE severity was graded according to National Cancer Institute's Common Toxicity Criteria (NCI‐CTC) version 4.0. An early skin reaction was defined as the worst severity of skin toxicity within 8 weeks from initiation of the treatment. The survey sheets, including safety, efficacy and compliance with treatment, were collected at registration and after 4, 8, 16, and 24 weeks.

QOL analyses were conducted in patients with a baseline and at least one postbaseline QOL assessment. QOL was assessed at baseline and after 2, 4, 8, 16, and 24 weeks, and a time window of 2 weeks around each follow‐up QOL assessment time point was accepted. If the patient did not complete the study treatment, the last QOL assessment was performed at the time of judgment of study termination or the nearest scheduled time point. The European Organisation for Research and Treatment of Cancer Quality of Life Questionnaire C30 (EORTC QLQ‐C30) version 3.0 was used to assess HRQOL because it is valid and reliable in the advanced cancer setting, including CRC.^21^, ^22^ This 30‐item questionnaire contains a global health status (GHS)/QOL scale, 5 functional scales, 3 symptom scales, and 6 single scales assessing additional symptoms.^21^ A difference of more than 10 points in change scores from baseline was considered clinically meaningful.^23^ The Dermatology Life Quality Index (DLQI), a widely validated skin‐specific self‐administered questionnaire,^8^ was used to assess skin‐related QOL. A change in DLQI score of at least 4 points was considered clinically meaningful.^24^ Questionnaire compliance rates were calculated as the number of patients who completed a questionnaire at a given time point divided by the number of patients expected to be evaluable at that time point.

### 2.3 Statistical analysis

Patients who withdrew consent before any intervention were excluded from all the analyses. In order to examine the impact of AEs on QOL, we analyzed the association of the worst grade of AEs with the changes in the EORTC QLQ‐C30 scores from baseline throughout observation period of 8 and 24 weeks. For this analysis, we used a linear mixed‐effects model for repeated measures, with the intercept and slope for the study week as random effects to estimate the least squared means of the change from baseline. The impact of skin toxicity on changes in DLQI scores and the impact of treatment efficacy on changes in EORTC QLQ‐C30 scores were also assessed using the same statistical analysis. The log‐rank test was used to compare the distribution of survival time. The association between time to event endpoints and early skin toxicity was analyzed using the Cox proportional hazard model, which calculates the adjusted hazard ratio (HR) and the 95% confidence interval (CI). All statistical analyses were conducted with the JMP 12 software package (SAS Institute, Cary, NC, USA).

Detailed methods are provided in the Supporting Information.

## 3 RESULTS

### 3.1 Overall population, treatment efficacy, and safety in this study

**Figure 1:**
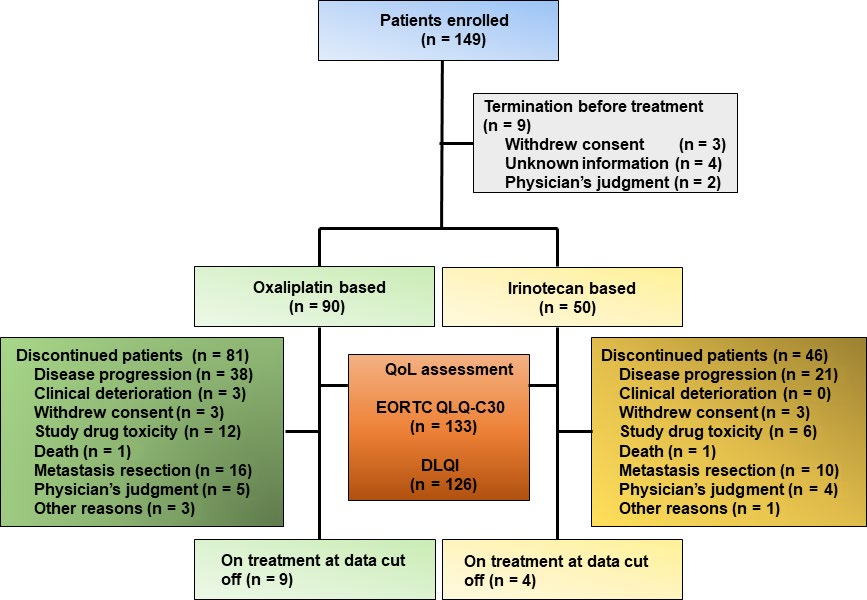
Patient disposition in the study. In total, 140 of 149 patients enrolled in the study received cetuximab plus chemotherapy, among which 133 and 127 were evaluable using the EORTC QLQ‐C30 and DLQI questionnaires, respectively

In total, 149 patients with KRAS wild‐type mCRC were enrolled from 49 institutions between July 2013 and April 2015. Nine patients were terminated from the study before the first administration of study treatment and 140 patients received cetuximab plus one of the standard chemotherapies; 90 (64.3%) were treated with mFOLFOX6 and 50 (35.7%) with FOLFIRI (Figure 1). The main reasons for treatment discontinuation were as follows: disease progression (46.5%), metastasectomy (20.4%), and treatment toxicity (14.2%). The baseline clinicopathological characteristics of the patients are outlined (Table 1). The data cutoff date was 20 April 2016, and by that date, 127 and 47 events were observed in relation to PFS and OS, respectively. The median duration of follow‐up time was 17.9 months (95% CI, 16.5‐19.1), and 72.8% of patients received subsequent chemotherapy after study termination. Median OS was not reached at the time of data cutoff, and the 2‐year estimated OS rate was 63.6%. The objective response rate (ORR) was 53.6% (95% CI, 45.3‐61.6), and the median PFS was 10.4 months (95% CI, 8.5‐11.8).

**Table 1:** Baseline clinicopathological characteristics

Characteristics	Number of patients (*n *=* *140)
Age (y)	
Median (range)	66 (27‐89)
Gender	
Female (%)	43 (30.7)
Male (%)	97 (69.3)
ECOG performance status (PS)	
PS0 (%)	111 (79.3)
PS1 (%)	26 (18.6)
PS2 (%)	3 (2.1)
GHS/QOL in EORTC QLQ‐C30	
Median (range)	58.3 (0‐100)
DLQI	
Median (range)	1.0 (0‐11)
Site of primary tumor	
Colon (%)	92 (65.7)
Rectum (%)	48 (34.3)
Histological differentiation	
Well/Moderate (%)	130 (92.9)
Poor (%)	6 (4.3)
Other (%)	4 (2.8)
Number of metastatic lesions	
1 (%)	54 (38.6)
≥2(%)	86 (61.4)
Metastatic sites	
Liver (%)	89 (63.6)
Liver only (%)	43 (30.7)
Lung (%)	33 (23.6)
Lymph node (%)	51 (36.4)
Other (%)	36 (25.7)
EGFR staining	
Negative (%)	3 (2.2)
Positive (%)	95 (68.8)
Unknown (%)	40 (29.0)
Previous treatment	
Surgery (%)	25 (19.8)
Adjuvant chemotherapy (%)	21 (15.1)

DLQI, Dermatology Life Quality Index; EORTC QLQ‐C30, European Organisation for Research and Treatment of Cancer Quality of Life Questionnaire Core 30; EGFR, epidermal growth factor receptor; GHS/QOL, global health status/quality of life.

The safety profile was consistent with the results from prior clinical trials (Table S1).^7^ Skin toxicity reactions, including acneiform exanthema, rash, dry skin, paronychia, and pruritus, occurred in most patients (91.4%). Skin toxicity reactions of grade 3 or higher were observed in 4.3% of the patients. Other cetuximab‐related AEs with a grade of ≥3 included infusion reaction, hypomagnesaemia, and interstitial lung disease, which occurred in 0.7%, 2.9%, and 4.3% of patients, respectively. The cetuximab dose was reduced in 27 patients (19.2%), and the mean relative dose intensity was 93.1%.

### 3.2 Association between skin toxicity and QOL in the EORTC QLQ‐C30 and DLQI questionnaires

In total, 133 and 126 of 140 patients were eligible for a HRQOL assessment by the EORTC QLQ‐C30 and skin‐related QOL assessment using the DLQI tools, respectively. Although the compliance rates of both questionnaires slightly declined over time, high compliance rates were maintained throughout the study period (i.e., 97.9% at baseline, 96.2% at 8 weeks, and 81.1% at 24 weeks for QLQ‐C‐30, and 92.9% at baseline, 90.6% at 8 weeks, and 78.7% at 24 weeks for DLQI). The median scores at baseline were 58.3 for GHS/QOL and 1.0 for DLQI, and there were no differences between the 2 different chemotherapy backbones. With regard to the preventive treatment for skin toxicity reactions, 123 of 140 patients (87.8%) received pre‐emptive skin treatment, including moisturizers, a topical steroid, and/or doxycycline.

**Figure 2:**
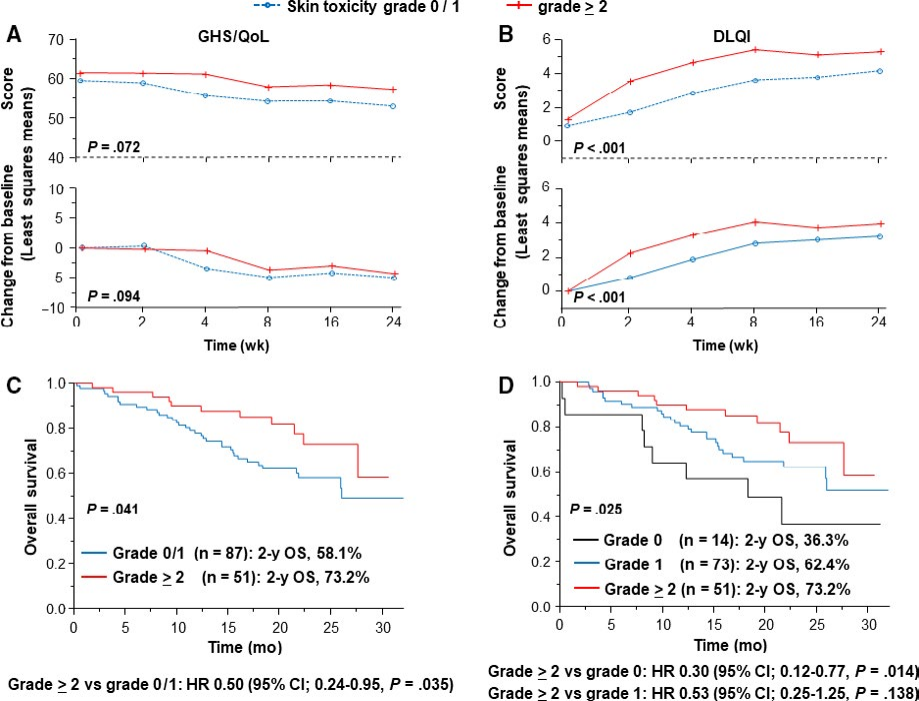
Association of the grade of skin toxicity reactions with QOL and treatment efficacy. A, Association between GHS/QOL and the worst grade of skin toxicity reactions (grade ≥ 2 vs grade 0 or 1) throughout observation period of 24 weeks. In total, 74 and 58 patients had grade ≥2 and grade 0 or 1 skin toxicity reactions, respectively. The least squares means of the GHS/QOL scores (upper graph) and of the changes from baseline (lower graph) at each time point. B, Association between skin‐related QOL and the worst grade of skin toxicity (grade ≥ 2 vs grade 0 or 1) throughout the study period. The least squares means of the DLQI scores (upper graph) and of the changes from baseline (lower graph) at each time point. C, The Kaplan‐Meier curves of OS according to the severity grades of early skin reaction (grade ≥2 vs grade 0 or 1). D, The Kaplan‐Meier curves of OS according to each grade of early skin reaction (grade ≥2 vs grade 1 vs grade 0)

The impact of early skin toxicity reactions in relation to the changes from baseline HRQOL and skin‐related QOL scores was estimated using a linear mixed‐effects model for repeated measures. The estimated mean changes from baseline in GHS/QOL to 8 weeks were −0.72 for patients without early skin reaction (grade 0) compared with −5.75 and −2.90 for those with a grade of 1 and ≥2, respectively. The differences were neither statistically significant nor clinically relevant (Table 2). Similarly, no significant change from the baseline was noted in each functioning scale (physical, role, cognitive, emotional, and social functioning scales). In addition, the worst grade of skin toxicity throughout the study period also showed no impact on GHS/QOL (Figure 2A). On the other hand, the worst skin toxicity, with a grade of ≥2, was significantly associated with an increased score and change from the baseline across all time points in DLQI, compared with a grade of 0 or 1 (*P *<* *.001) (Figure 2B). The mean score peaked at 8 weeks, changing from 1.38 at baseline to 5.13 in patients with a grade of ≥2. The difference between scores from baseline to 8 weeks was less than 4.0 points, indicating no clinically relevant impairment.^24^ Thus, with respect to skin toxicity reactions, there was no critical impact on either HRQOL or skin‐related QOL of the patients.

**Table 2:** Change from baseline in HRQOL at 8 wk stratified by severity grades of early skin reaction

EORTC QLQ‐C30 dimensions at 8 wk	Early skin reaction (8 wk)^a^		
	Grade 0	Grade 1	Grade ≥ 2
GHS/QOL			
Number of patients	9	66	48
LSM ± SEM	−0.72 ± 7.23	−5.75 ± 2.78	−2.90 ± 3.28
*P*‐value^b^	—	.75	.94
*P*‐value^c^	—	—	.71
Social functioning			
Number of patients	9	68	48
LSM ± SEM	1.83 ± 7.04	−2.06 ± 2.61	2.56 ± 3.14
*P*‐value^b^	—	.89	.64
*P*‐value^c^	—	—	.25
Physical functioning			
Number of patients	9	68	47
LSM ± SEM	5.56 ± 15.59	0.62 ± 6.09	−5.91 ± 7.28
*P*‐value^b^	—	.92	.53
*P*‐value^c^	—	—	.28
Role functioning			
Number of patients	9	68	48
LSM ± SEM	6.18 ± 8.66	−7.68 ± 3.34	−8.71 ± 3.40
*P*‐value^b^	—	.30	.24
*P*‐value^c^	—	—	.57
Cognitive functioning			
Number of patients	9	68	48
LSM ± SEM	−2.83 ± 7.23	1.83 ± 7.04	1.83 ± 7.04
*P*‐value^b^	—	.51	.47
*P*‐value^c^	—	—	.76
Emotional functioning			
Number of patients	9	68	48
LSM ± SEM	8.92 ± 5.61	1.51 ± 2.14	4.97 ± 2.55
*P*‐value^b^	—	.22	.46
*P*‐value^c^	—	—	.56

EORTC QLQ‐C30, European Organisation for Research and Treatment of Cancer Quality of Life Questionnaire Core 30; GHS/QOL, global health status/Quality of Life; LSM, least squares mean; SEM, standard error of mean.

a The worst grades of skin toxicity during the first 8 weeks. Grades were determined according to the National Cancer Institute Common Toxicity Criteria, version 4.0.

b *P*‐value between grade 0 and skin toxicity (grade 1 or grade ≥2).

c *P*‐value between grade 0/1 and grade ≥2 (liner mixed‐effect model).

### 3.3 Early skin reaction and treatment efficacy

An early skin reaction with a grade of ≥2 showed a significantly favorable OS compared with a grade of 0 or 1 (HR, 0.50; 95% CI, 0.24‐0.95; *P *=* *.035) (Figure 2C), and a significant overall survival advantage was correlated to the early skin reaction grade (2‐year OS rates, 73.2% for grade ≥2, 62.4% for grade 1, 36.3% for grade 0, *P *=* *.025) (Figure 2D). Similar findings were observed even when patients (*n *=* *11) were excluded due to early termination, including death or clinical progression of the disease, within 8 weeks of starting treatment. Furthermore, an early skin reaction remained an independent predictor of OS (HR, 0.48; 95% CI, 0.21‐0.97; *P *=* *.040) in the multivariable Cox proportional hazard model after adjustment with respect to demographic and disease characteristics as well as study and follow‐up treatments by the considering covariates pre‐emptive skin treatment, age, gender, an Eastern Cooperative Oncology Group Performance Status (ECOG PS) score, chemotherapy backbone, site of primary tumor, presence of primary tumor, number of metastatic lesions, metastatic sites (liver only vs other), and second‐line chemotherapy (Table 3).

**Table 3:** Univariate and multivariable prognostic analyses were performed using the Cox proportional hazard model

Variables	Univariate			Multivariable		
	HR	95% CI	*P‐*value^a^	HR	95% CI	*P‐*value^a^
Early skin reaction						
Grade ≥ 2 vs grade 0 or 1	0.50	0.24‐0.95	**.035**	0.48	0.21‐0.97	**.040**
Second‐line chemotherapy						
Presence vs absence	0.50	0.74‐2.63	**.036**	0.46	0.22‐0.97	**.042**
Age						
Age ≥70 vs <70 (y)	2.07	1.16‐3.73	**.014**	1.97	1.03‐3.77	**.042**
ECOG PS						
PS ≥1 vs PS 0	2.55	1.35‐4.67	**.005**	1.59	0.71‐3.39	.253
Gender						
Male vs female	1.34	0.71‐2.70	.373	1.39	0.68‐3.05	.375
Chemotherapy backbone						
mFOLFOX6 vs FOLFIRI	1.36	0.74‐2.63	.327	1.16	0.53‐2.42	.702
Pre‐emptive skin treatment						
Presence vs absence	1.09	0.47‐3.15	.856	1.06	0.51‐2.16	.877
Site of primary tumor						
Colon vs rectum	1.58	0.30‐1.21	.174	1.48	0.71‐3.35	.302
Primary tumor						
Presence vs absence	1.47	0.80‐2.63	.206	1.14	0.60‐2.26	.720
Number of metastatic lesions						
1 vs ≥2	0.84	0.44‐1.52	.561	1.06	0.51‐2.16	.877
Metastatic sites						
Liver only vs the other	0.58	0.32‐1.04	.068	0.61	0.84‐3.24	.145

PS, Performance status.

Cox proportional hazard model.

Bold values show statistical significance (*P* < .05).

### 3.4 Changes in HRQOL based on the status of baseline tumor‐related symptoms and tumor response

To evaluate the association between QOL and treatment efficacy based on the status of tumor‐related symptoms at baseline, changes in QOL according to tumor response at 8 weeks were assessed in subgroups of patients with and without symptoms at baseline. Patients were considered symptomatic if they answered “quite a bit” or “very much” to at least one of the symptom questions of EORTC QLQ‐C30 at baseline and asymptomatic if they answered “not at all” or “a little” to all of the symptoms.

Compared to nonresponders, response to treatment was associated with an improved HRQOL within 8 weeks in symptomatic patients and decreased deterioration in HRQOL among asymptomatic patients (Figure S1a). At the 8‐week time point, symptomatic responders experienced statistically significant and clinically meaningful improvements in role functioning (mean change score, +12.75; *P *=* *.015) and social functioning (mean change score, +15.52; *P *<* *.001), compared to symptomatic nonresponders (Figure S1b). In asymptomatic patients, the GHS/QOL score deteriorated to a clinically meaningful degree at 8 weeks in nonresponders (mean change score, −13.39), while it was maintained throughout the study period in responders (mean change score, −5.04 at 8 weeks and −6.85 at 24 weeks).

### 3.5 Association between adverse events and QOL in chemotherapy plus cetuximab

**Figure 3:**
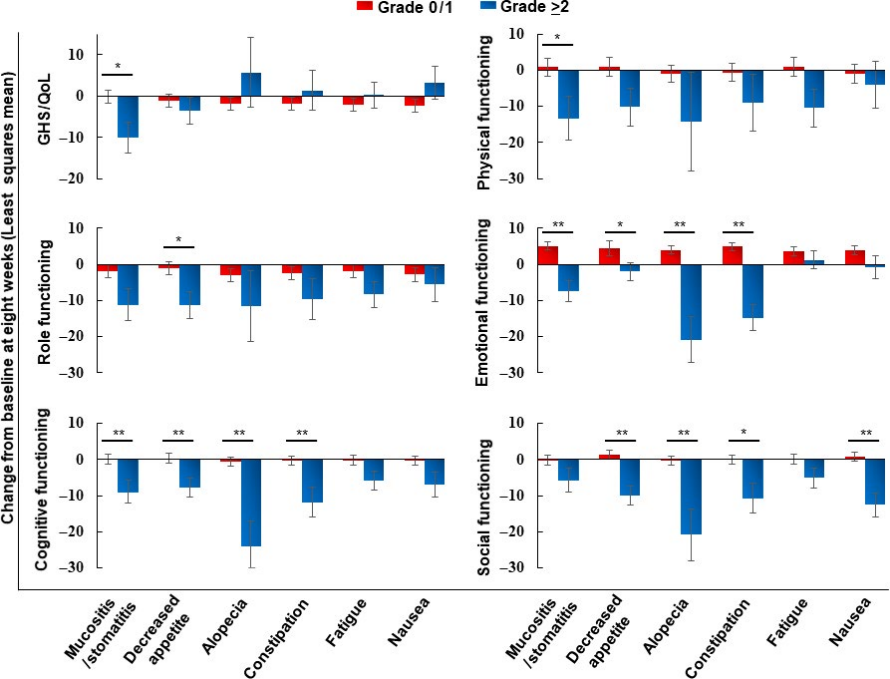
Changes from baseline in HRQOL according to the worst grades (grade ≥2 vs grade 0 or 1) of AEs during the first 8 wk. There were 20 patients with grade ≥2 and 113 with grade 0 or 1 mucositis/stomatitis, 27 with grade ≥2 and 106 with grade 0 or 1 decreased appetite, 4 with grade ≥2 and 129 with grade 0 or 1 alopecia, 12 with grade ≥2 and 121 with grade 0 or 1 constipation, 28 with grade ≥2 and 105 with grade 0 or 1 fatigue, and 18 with grade ≥2 and 115 with grade 0 or 1 nausea. *P <* *.05; **P <* *.01 (linear mixed‐effects model), bars; mean ± SEM

It remains unclear which AEs place patients at risk for clinically relevant HRQOL declines following cetuximab containing regimens. The worst grades of common AEs throughout the study period were concomitantly assessed using a linear mixed‐effects model for repeated measures. Exploratory comparisons of HRQOL and AEs revealed statistically significant and clinically meaningful differences (Table S2). Among those AEs, mucositis/stomatitis was associated with the worsening of mean GHS/QOL scores (−12.64 for grade ≥2 vs −0.35 for grade 0 or 1, *P *=* *.005), physical functioning (−15.10 for grade ≥2 vs −1.28 for grade 0 or 1, *P *=* *.016), and role functioning (−16.11 for grade ≥2 vs −2.61 for grade 0 or 1, *P *=* *.008). Other AEs that impaired functional well‐being include decreased appetite, alopecia, constipation, fatigue, nausea, and vomiting. These AEs also showed similar findings at the 8‐week time point (Figure 3). As expected, the relative dose intensity of the regimen was significantly decreased in patients with ≥grade 2 mucositis/stomatitis compared to those with ≤grade 1 symptoms (87.8% vs 94.1% for cetuximab, *P *=* *.008).

## 4 DISCUSSION

In recent clinical trials, to bridge the considerable gap between perceptions of patients and physicians regarding the toxicity during of treatments, there has been an increasing interest and emphasis on the inclusion of QOL as one of the substantial key components to determine the success of cancer therapy.^6^ At the scene of ordinary clinical practice, a balance between the expected benefit and the possible risk of a detriment of QOL should be carefully considered. This attitude is especially important in the treatment of patients with unresectable mCRC, as treatment aims are generally palliative rather than curative.^2^

Several retrospective analyses of large randomized trial using anti‐EGFR ab plus standard chemotherapy for RAS wild‐type mCRC have reported that the appearance of severe skin toxicity reactions is associated with better survival outcomes.^8^, ^12^, ^13^ However, these observations might be confounded by differences in treatment exposure, because responding patients were likely to undergo a longer duration of treatment, leading to greater cumulative toxicity as well as the better prognosis. Although these findings seem to be convincing, “ad hoc” analyses have potential bias due to their retrospective nature.

The aim of our present study was to examine and confirm the findings obtained from the previous exploratory analyses in a newly designed prospective predefined clinical trial, investigating whether (1) skin toxicity severity from cetuximab is predictive of better treatment outcomes in patients with RAS wild‐type mCRC; (2) skin toxicity reactions are or are not related to the deterioration of total HRQOL of the patients; and (3) what is the most substantial dose limiting toxicity of cetuximab containing regimen that affects HRQOL and may lead to the discontinuation of the treatment?

In order to avoid confounding factors in this study, the association between the treatment outcomes and skin toxicity reactions was assessed using the worst grade of skin toxicity severity during the first 8 weeks that is the first scheduled time point of radiological assessment of treatment response. This time point was expected not only to minimize the influence of early study termination due to the first radiological assessment, but also to elicit the largest influence on QOL.^25^ Our findings from this prospective study confirm the previous exploratory analyses, demonstrating the association between early skin reactions and favorable outcomes.

With regard to the second clinical question, whether skin toxicity reactions cause a deterioration of patient QOL, the association between skin toxicity reactions and HRQOL was evaluated. This study prospectively demonstrated that skin toxicity had no clinical impact on HRQOL or skin‐related QOL. Skin toxicity reactions of grade 3 were observed in 4.3% of the patients, and the incidence rate was lower than that of previous clinical trials using anti‐EGFR ab plus chemotherapy without pre‐emptive skin treatment (a grade of ≥3 ranged from 12.9% to 26.0%).^7^, ^26^, ^27^, ^28^ Our present study adopted prophylactic skin treatment. The STEPP trial assessing the efficacy of pre‐emptive skin treatment demonstrated that prophylactic skin treatment could reduce more than half of the severity of skin toxicity reactions during treatment with anti‐EGFR ab, similar to the incidence rate of grade 3 skin toxicity (6.3%).^29^ A deterioration of QOL was observed among patients with grade 3 skin toxicity reactions, but not those with a grade of 2: the estimated mean changes from baseline throughout the study period in GHS/QOL and DLQI were −28.42 and 6.92 for patients with a grade of 3 compared with −2.04 and 3.68 for those with a grade of 2, respectively. Thus, in ordinary clinical practice based on the guidelines for the prevention and treatment of anti‐EGFR ab‐related dermatologic toxicities,^30^ the alleviation of grade 3 skin toxicity reactions by prophylactic management might result in no impairment of either HRQOL or skin‐related QOL.

With respect to the third clinical question, influence of AEs other than skin toxicity reactions on HRQOL was also examined by our planned analysis. The addition of cetuximab to the standard chemotherapy for mCRC sometimes exacerbates AEs, including mucositis/stomatitis, decreased appetite, or diarrhea.^17^, ^19^ However, lack of data regarding the impact of these AEs on HRQOL has hindered personalized decision‐making based on the patient's perspective. The present study demonstrated that mucositis/stomatitis is a clinically relevant detrimental factor impacting HRQOL, both throughout observation period of 8 and 24 weeks. Mucositis/stomatitis is a common AE of cancer therapy,^31^ and the addition of cetuximab enhances chemotherapy‐induced mucositis/stomatitis independent of the chemotherapy backbone via the inhibition of the regenerative and protective effects of the healing process.^17^ Of note, mucositis/stomatitis of grade ≥2 will lead to a lower relative dose intensity. Therefore, severe mucositis/stomatitis may deteriorate both QOL and treatment compliance, highlighting the paramount importance of its timely and appropriate management.

The present study is limited by its relatively small sample size, which hampered part of the statistical analyses. It was not possible to fully determine the influence of objective clinical factors on HRQOL over time. This study did not include a control group who received another standard regimen, such as bevacizumab plus chemotherapy; thus, the interpretation of findings was limited to a comparison of outcomes between subgroups in this study population, where all the patients received cetuximab plus chemotherapy treatment. A possible selection bias cannot be excluded because the choice of the chemotherapy backbone (FOLFIRI or FOLFOX) was under at the discretion of individual participating physician. It also remains unclear how differences in race, ethnicity, and physical activity may have affected HRQOL. Thus, these limitations should be taken into account when interpreting and generalizing the results of this study. On the other hand, the strengths of this study are the prospective nature of its design, the higher rate of questionnaire completion throughout the study compared with previous reports,^10^, ^32^ and the use of both well‐established global and skin‐specific QOL questionnaire surveys. Future studies are required to build on the findings of this study.

In conclusion, this study provides novel insights into the association of the treatment effect, disease symptoms, and AEs with QOL during treatment with cetuximab plus chemotherapy. These data may be clinically useful for physicians and patients, improving their understanding of expected outcomes and enhancing their ability to make more properly informed decisions.

## CONFLICT OF INTEREST

Merck KGaA reviewed the manuscript for medical accuracy only before journal submission. Detailed conflict of interest disclosures for each author are provided in the Supporting Information.
