# Supplementary material for: A prospective Phase II study to examine the relationship between quality of life and adverse events of first‐line chemotherapy plus cetuximab in patients with KRAS wild‐type unresectable metastatic colorectal cancer: QUACK trial
Source: Cancer Med. 2018 Jul 26;7(9):4217–27. doi: 10.1002/cam4.1623 (PMC6144158; doi:10.1002/cam4.1623)
Supplement: Supplementary file 1 [file CAM4-7-4217-s001.docx]

Supplementary Figure S1

**
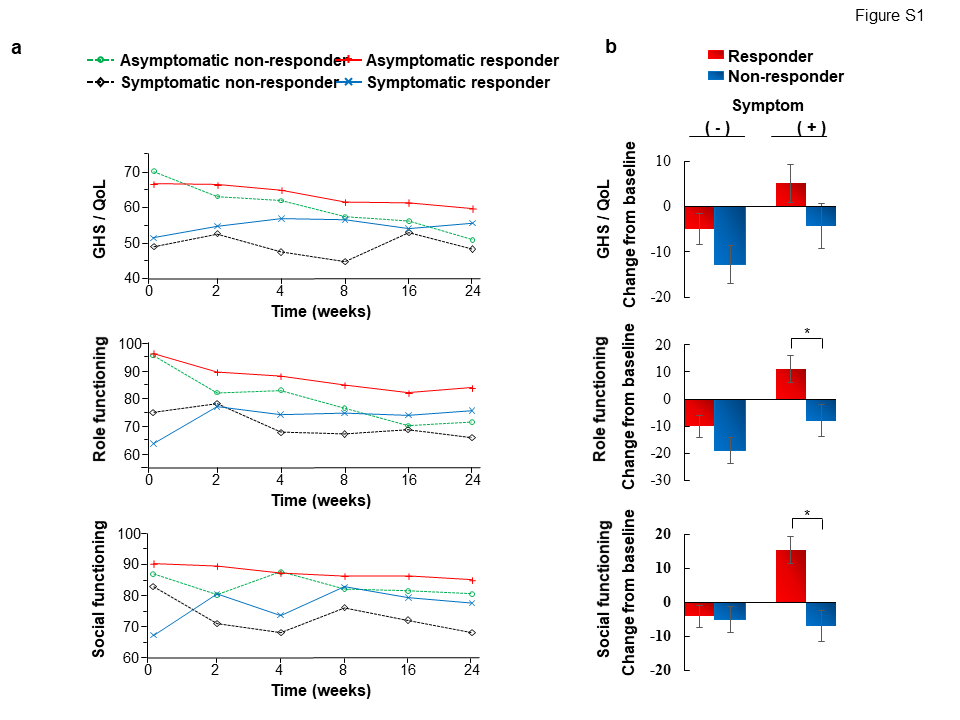
**

**Supplementary Figure S1**: Changes in HRQOL according to treatment response in patients with or without symptoms at baseline. **a**) The least squares means of the GHS/QOL and functioning scales at each time point in asymptomatic non-responders (*n* = 33), asymptomatic responders (*n* = 44), symptomatic non-responders (*n* = 23), and symptomatic responders (*n* = 29). **b**) The least squares means of the changes from baseline in the GHS/QOL and functioning scales at eight weeks. *, *P* < 0.05 (difference between responder and non-responder according to the symptom at baseline by linear mixed-effects model).

Bars; the mean + SEM.
