# Supplementary material for: A prospective Phase II study to examine the relationship between quality of life and adverse events of first‐line chemotherapy plus cetuximab in patients with KRAS wild‐type unresectable metastatic colorectal cancer: QUACK trial
Source: Cancer Med. 2018 Jul 26;7(9):4217–27. doi: 10.1002/cam4.1623 (PMC6144158; doi:10.1002/cam4.1623)
Supplement: Supplementary file 2 [file CAM4-7-4217-s002.docx]

| \| **Supplementary Table S1. Most common adverse events (*n* = 140)** \| \| \| \| --- \| --- \| --- \| \| **Event** \| **Any grade (%)*** \| **Grade >3 (%)*** \| \| Any adverse event \| 139 (99.3) \| 74 (52.9) \| \| Skin toxicity† \| 128 (91.4) \| 6 (4.3) \| \| Acneiform exanthema/rash \| 119 (85.0) \| 5 (3.6) \| \| Dry skin \| 90 (64.3) \| 2 (1.4) \| \| Paronychia \| 75 (53.6) \| 0 (0.0) \| \| Pruritus \| 55 (39.3) \| 0 (0.0) \| \| Mucositis/stomatitis \| 71 (50.7) \| 2 (1.4) \| \| Fatigue \| 80 (57.1) \| 6 (4.3) \| \| Anorexia \| 78 (55.7) \| 8 (5.7) \| \| Diarrhea \| 48 (34.3) \| 4 (2.9) \| \| Nausea \| 53 (37.9) \| 2 (1.4) \| \| Vomitting \| 27 (19.3) \| 3 (2.1) \| \| Constipation \| 44 (31.4) \| 0 (0.0) \| \| Alopecia \| 32 (22.9) \| 0 (0.0) \| \| Peripheral neuropathy \| 81 (57.9) \| 2 (1.4) \| \| Haematotoxicity† \| 126 (90.0) \| 46 (32.9) \| \| Neutropenia‡ \| 84 (60.0) \| 45 (32.1) \| \| Electrolyte imbalance† \| 109 (77.9) \| 20 (14.3) \| \| Liver toxicity† \| 86 (61.4) \| 5 (3.6) \| \| Nephrotoxicity† \| 73 (52.1) \| 3 (2.1) \| \| Special adverse events \|  \|  \| \| Infusion-related reaction \| 5 (3.6) \| 1 (0.7) \| \| Hypomagnesemia \| 59 (42.1) \| 4 (2.9) \| \| Interstitial lung disease§ \| 8 (5.7) \| 6 (4.3) \| |
| --- | --- | --- | --- | --- | --- | --- | --- | --- | --- | --- | --- | --- | --- | --- | --- | --- | --- | --- | --- | --- | --- | --- | --- | --- | --- | --- | --- | --- | --- | --- | --- | --- | --- | --- | --- | --- | --- | --- | --- | --- | --- | --- | --- | --- | --- | --- | --- | --- | --- | --- | --- | --- | --- | --- | --- | --- | --- | --- | --- | --- | --- | --- | --- | --- | --- | --- | --- | --- | --- | --- | --- | --- | --- | --- | --- | --- | --- | --- |

*Grades were determined accoding to the National Cancer Institute Common Toxicity Criteria, version 4.0.

†Composite categories; ‡Grade 3 or 4 febrile neutropenia was reported in 8 patients;

§Grade 5 adverse event was reported in 1 patient
