# Supplementary material for: A prospective Phase II study to examine the relationship between quality of life and adverse events of first‐line chemotherapy plus cetuximab in patients with KRAS wild‐type unresectable metastatic colorectal cancer: QUACK trial
Source: Cancer Med. 2018 Jul 26;7(9):4217–27. doi: 10.1002/cam4.1623 (PMC6144158; doi:10.1002/cam4.1623)
Supplement: Supplementary file 3 [file CAM4-7-4217-s003.docx]

| \| **Supplementary Table S2. Change from baseline throughout the study in GHS/QoL and functional scales stratified by severity levels of common adverse events** \| \| \| \| \| \| \| \| \| \| \| \| \| \| --- \| --- \| --- \| --- \| --- \| --- \| --- \| --- \| --- \| --- \| --- \| --- \| --- \| \|  \| **GHS/QoL** \| \| **Physical** \| \| **Role** \| \| **Emotional** \| \| **Cognitive** \| \| **Social** \| \| \| **Adverse event*** \| **LSM + SEM** \| ***P*-value†** \| **LSM + SEM** \| ***P*-value†** \| **LSM + SEM** \| ***P*-value†** \| **LSM + SEM** \| ***P*-value†** \| **LSM + SEM** \| ***P*-value†** \| **LSM + SEM** \| ***P*-value†** \| \| Mucositis/stomatitis \|  \| .005 \|  \| .016 \|  \| .008 \|  \| < .001 \|  \| .028 \|  \| .015 \| \| Grade 0/1 \| -0.35 + 1.76 \|  \| -1.28 + 2.37 \|  \| -2.61 + 2.05 \|  \| 5.60 + 1.34 \|  \| -1.40 + 1.42 \|  \| 0.24 + 1.49 \|  \| \| Grade >2 \| -12.64 + 3.92 \|  \| -15.10 + 5.15 \|  \| -16.11 + 4.56 \|  \| -7.53 + 2.97 \|  \| -9.07 + 3.15 \|  \| -8.70 + 3.29 \|  \| \| Decreased appetite \|  \| .322 \|  \| .014 \|  \| .004 \|  \| .008 \|  \| .001 \|  \| < .001 \| \| Grade 0/1 \| -1.61 + 1.87 \|  \| -0.85 + 2.45 \|  \| -2.00 + 2.14 \|  \| 5.03 + 1.44 \|  \| -0.47 + 1.45 \|  \| 1.28 + 1.52 \|  \| \| Grade >2 \| -5.44 + 3.38 \|  \| -13.61 + 4.50 \|  \| -14.75 + 3.84 \|  \| -2.92 + 2.60 \|  \| -10.50 + 2.62 \|  \| -10.28 + 2.75 \|  \| \| Alopecia \|  \| .238 \|  \| .171 \|  \| .423 \|  \| < .001 \|  \| .016 \|  \| .031 \| \| Grade 0/1 \| -2.99 + 1.69 \|  \| -3.01 + 2.25 \|  \| -4.54 + 1.96 \|  \| 4.43 + 1.26 \|  \| -1.96 + 1.33 \|  \| -0.65 + 1.41 \|  \| \| Grade >2 \| 5.21 + 6.71 \|  \| -15.87 + 9.05 \|  \| -11.11 + 7.94 \|  \| -15.67 + 5.16 \|  \| -15.48 + 5.41 \|  \| -13.49 + 5.72 \|  \| \| Constipation \|  \| .669 \|  \| .243 \|  \| .121 \|  \| < .001 \|  \| .003 \|  \| .011 \| \| Grade 0/1 \| -2.72 + 1.73 \|  \| -2.86 + 2.30 \|  \| -3.86 + 1.99 \|  \| 5.55 + 1.22 \|  \| -1.39 + 1.34 \|  \| -0.13 + 1.43 \|  \| \| Grade >2 \| -0.32 + 5.31 \|  \| -11.40 + 6.90 \|  \| -13.78 + 6.05 \|  \| -16.52 + 3.74 \|  \| -14.51 + 4.10 \|  \| -11.90 + 4.34 \|  \| \| Fatigue \|  \| .622 \|  \| .027 \|  \| .059 \|  \| .287 \|  \| .005 \|  \| .064 \| \| Grade 0/1 \| -2.08 + 1.88 \|  \| -1.22 + 2.44 \|  \| -3.07 + 2.16 \|  \| 3.98 + 1.46 \|  \| -0.82 + 1.47 \|  \| 0.03 + 1.57 \|  \| \| Grade >2 \| -4.01 + 3.43 \|  \| -12.91 + 4.63 \|  \| -11.67 + 3.97 \|  \| 0.72 + 2.68 \|  \| -9.47 + 2.68 \|  \| -6.08 + 2.86 \|  \| \| Nausea \|  \| .312 \|  \| .566 \|  \| .468 \|  \| .079 \|  \| .109 \|  \| < .001 \| \| Grade 0/1 \| -3.17 + 1.77 \|  \| -3.21 + 2.38 \|  \| -4.63 + 2.06 \|  \| 4.27 + 1.37 \|  \| -1.84 + 1.41 \|  \| 0.70 + 1.44 \|  \| \| Grade >2 \| 1.55 + 4.29 \|  \| -6.73 + 5.66 \|  \| -8.20 + 4.94 \|  \| -2.08 + 3.31 \|  \| -7.78 + 3.40 \|  \| -12.61 + 3.45 \|  \| \| Vomitting \|  \| .112 \|  \| .187 \|  \| .300 \|  \| .218 \|  \| .154 \|  \| .039 \| \| Grade 0/1 \| -3.18 + 1.68 \|  \| -4.54 + 2.24 \|  \| -4.38 + 1.97 \|  \| 3.76 + 1.32 \|  \| -2.21 + 1.34 \|  \| -0.54 + 1.42 \|  \| \| Grade >2 \| 7.32 + 6.34 \|  \| 6.82 + 8.27 \|  \| -12.43 + 7.59 \|  \| -2.56 + 4.93 \|  \| -9.67 + 5.04 \|  \| -11.88 + 5.26 \|  \| \| Peripheral neuropathy \|  \| .865 \|  \| .464 \|  \| .555 \|  \| .009 \|  \| .160 \|  \| .254 \| \| Grade 0/1 \| -2.51 + 1.89 \|  \| -2.77 + 2.54 \|  \| -4.22 + 2.19 \|  \| 5.42 + 1.44 \|  \| -1.63 + 1.50 \|  \| -0.35 + 1.59 \|  \| \| Grade >2 \| -1.84 + 3.44 \|  \| -6.54 + 4.47 \|  \| -6.89 + 3.95 \|  \| -2.54 + 2.61 \|  \| -6.02 + 2.72 \|  \| -4.30 + 2.89 \|  \| \| Diarrhea \|  \| .227 \|  \| .251 \|  \| .682 \|  \| .036 \|  \| .095 \|  \| .611 \| \| Grade 0/1 \| -3.12 + 1.73 \|  \| -2.91 + 2.31 \|  \| -4.66 + 2.02 \|  \| 4.19 + 1.34 \|  \| -2.02 + 1.37 \|  \| -1.12 + 1.47 \|  \| \| Grade >2 \| 3.37 + 5.06 \|  \| -11.26 + 6.87 \|  \| -7.21 + 5.88 \|  \| -4.60 + 3.93 \|  \| -9.18 + 4.03 \|  \| -3.44 + 4.30 \|  \| \| Haematotoxicity‡ \|  \| .280 \|  \| .947 \|  \| .518 \|  \| .184 \|  \| .672 \|  \| .518 \| \| Grade 0/1 \| -4.91 + 2.74 \|  \| -3.96 + 3.69 \|  \| -6.59 + 3.17 \|  \| 5.62 + 2.14 \|  \| -2.00 + 2.20 \|  \| -0.15 + 2.32 \|  \| \| Grade >2 \| -1.19 + 2.06 \|  \| -3.65 + 2.73 \|  \| -4.02 + 2.39 \|  \| 2.06 + 1.60 \|  \| -3.16 + 1.64 \|  \| -2.03 + 1.73 \|  \| \| Neutropenia \|  \| .179 \|  \| .984 \|  \| .488 \|  \| .707 \|  \| .729 \|  \| .849 \| \| Grade 0/1 \| -4.84 + 2.33 \|  \| -3.72 + 3.16 \|  \| -3.62 + 2.69 \|  \| 3.85 + 1.83 \|  \| -3.21 + 1.87 \|  \| -1.60 + 1.98 \|  \| \| Grade >2 \| -0.40 + 2.31 \|  \| -3.81 + 3.07 \|  \| -6.27 + 2.70 \|  \| 2.88 + 1.81 \|  \| -2.30 + 1.85 \|  \| -1.07 + 1.95 \|  \| \| Electrolyte imbalance‡ \|  \| .972 \|  \| .943 \|  \| .205 \|  \| .686 \|  \| .385 \|  \| .059 \| \| Grade 0/1 \| -2.43 + 1.87 \|  \| -3.73 + 2.50 \|  \| -6.17 + 2.15 \|  \| 3.56 + 1.46 \|  \| -2.14 + 1.49 \|  \| 0.08 + 1.56 \|  \| \| Grade >2 \| -2.57 + 3.45 \|  \| -4.10 + 4.69 \|  \| -0.39 + 4.00 \|  \| 2.32 + 2.69 \|  \| -4.87 + 2.77 \|  \| -6.17 + 2.89 \|  \| \| Hypomagnesemia \|  \| .150 \|  \| .390 \|  \| .883 \|  \| .421 \|  \| .683 \|  \| .798 \| \| Grade 0/1 \| -1.73 + 1.71 \|  \| -4.40 + 2.29 \|  \| -4.82 + 1.99 \|  \| 2.96 + 1.35 \|  \| -2.60 + 1.38 \|  \| -1.26 + 1.45 \|  \| \| Grade >2 \| -10.13 + 5.55 \|  \| 2.44 + 7.61 \|  \| -5.83 + 6.52 \|  \| 6.63 + 4.35 \|  \| -4.54 + 4.53 \|  \| -2.54 + 4.76 \|  \| \| Liver toxicity‡ \|  \| .306 \|  \| .413 \|  \| .752 \|  \| .294 \|  \| .373 \|  \| .154 \| \| Grade 0/1 \| -1.80 + 1.78 \|  \| -3.03 + 2.36 \|  \| -4.68 + 2.07 \|  \| 3.82 + 1.38 \|  \| -2.24 + 1.42 \|  \| -0.53 + 1.50 \|  \| \| Grade >2 \| -6.56 + 4.28 \|  \| -8.20 + 5.84 \|  \| -6.39 + 4.99 \|  \| 0.03 + 3.31 \|  \| -5.54 + 3.41 \|  \| -6.08 + 3.57 \|  \| \| Nephrotoxicity‡ \|  \| .248 \|  \| .730 \|  \| .281 \|  \| .251 \|  \| .875 \|  \| .460 \| \| Grade 0/1 \| -1.91 + 1.72 \|  \| -3.49 + 2.31 \|  \| -4.29 + 1.99 \|  \| 3.75 + 1.35 \|  \| -2.67 + 1.38 \|  \| -1.00 + 1.46 \|  \| \| Grade >2 \| -8.38 + 5.31 \|  \| -6.19 + 7.48 \|  \| -11.46 + 6.32 \|  \| -1.25 + 4.12 \|  \| -3.37 + 4.23 \|  \| -4.48 + 4.47 \|  \| |
| --- | --- | --- | --- | --- | --- | --- | --- | --- | --- | --- | --- | --- | --- | --- | --- | --- | --- | --- | --- | --- | --- | --- | --- | --- | --- | --- | --- | --- | --- | --- | --- | --- | --- | --- | --- | --- | --- | --- | --- | --- | --- | --- | --- | --- | --- | --- | --- | --- | --- | --- | --- | --- | --- | --- | --- | --- | --- | --- | --- | --- | --- | --- | --- | --- | --- | --- | --- | --- | --- | --- | --- | --- | --- | --- | --- | --- | --- | --- | --- | --- | --- | --- | --- | --- | --- | --- | --- | --- | --- | --- | --- | --- | --- | --- | --- | --- | --- | --- | --- | --- | --- | --- | --- | --- | --- | --- | --- | --- | --- | --- | --- | --- | --- | --- | --- | --- | --- | --- | --- | --- | --- | --- | --- | --- | --- | --- | --- | --- | --- | --- | --- | --- | --- | --- | --- | --- | --- | --- | --- | --- | --- | --- | --- | --- | --- | --- | --- | --- | --- | --- | --- | --- | --- | --- | --- | --- | --- | --- | --- | --- | --- | --- | --- | --- | --- | --- | --- | --- | --- | --- | --- | --- | --- | --- | --- | --- | --- | --- | --- | --- | --- | --- | --- | --- | --- | --- | --- | --- | --- | --- | --- | --- | --- | --- | --- | --- | --- | --- | --- | --- | --- | --- | --- | --- | --- | --- | --- | --- | --- | --- | --- | --- | --- | --- | --- | --- | --- | --- | --- | --- | --- | --- | --- | --- | --- | --- | --- | --- | --- | --- | --- | --- | --- | --- | --- | --- | --- | --- | --- | --- | --- | --- | --- | --- | --- | --- | --- | --- | --- | --- | --- | --- | --- | --- | --- | --- | --- | --- | --- | --- | --- | --- | --- | --- | --- | --- | --- | --- | --- | --- | --- | --- | --- | --- | --- | --- | --- | --- | --- | --- | --- | --- | --- | --- | --- | --- | --- | --- | --- | --- | --- | --- | --- | --- | --- | --- | --- | --- | --- | --- | --- | --- | --- | --- | --- | --- | --- | --- | --- | --- | --- | --- | --- | --- | --- | --- | --- | --- | --- | --- | --- | --- | --- | --- | --- | --- | --- | --- | --- | --- | --- | --- | --- | --- | --- | --- | --- | --- | --- | --- | --- | --- | --- | --- | --- | --- | --- | --- | --- | --- | --- | --- | --- | --- | --- | --- | --- | --- | --- | --- | --- | --- | --- | --- | --- | --- | --- | --- | --- | --- | --- | --- | --- | --- | --- | --- | --- | --- | --- | --- | --- | --- | --- | --- | --- | --- | --- | --- | --- | --- | --- | --- | --- | --- | --- | --- | --- | --- | --- | --- | --- | --- | --- | --- | --- | --- | --- | --- | --- | --- | --- | --- | --- | --- | --- | --- | --- | --- | --- | --- | --- | --- | --- | --- | --- | --- | --- | --- | --- | --- | --- | --- | --- | --- | --- | --- | --- | --- | --- | --- | --- | --- | --- | --- | --- | --- | --- | --- | --- | --- | --- | --- | --- | --- | --- | --- | --- | --- | --- | --- | --- | --- | --- | --- | --- | --- | --- | --- | --- | --- | --- | --- | --- | --- | --- | --- | --- | --- | --- | --- | --- | --- | --- | --- | --- | --- | --- | --- | --- | --- | --- | --- | --- | --- | --- | --- | --- | --- | --- | --- | --- | --- | --- | --- | --- | --- | --- | --- | --- | --- | --- | --- | --- | --- | --- | --- | --- | --- | --- | --- | --- | --- | --- | --- | --- | --- | --- | --- | --- | --- | --- | --- | --- | --- | --- | --- | --- | --- | --- | --- | --- | --- | --- | --- | --- | --- | --- | --- | --- | --- | --- | --- | --- | --- | --- | --- | --- | --- | --- | --- | --- | --- | --- | --- | --- | --- | --- | --- | --- | --- | --- | --- | --- | --- | --- | --- | --- | --- | --- | --- | --- | --- | --- | --- | --- | --- | --- | --- | --- | --- | --- | --- | --- | --- | --- | --- | --- | --- | --- | --- | --- | --- | --- | --- | --- | --- | --- | --- | --- | --- | --- | --- | --- | --- | --- | --- | --- | --- | --- | --- | --- | --- | --- | --- |

Abbreviations: LSM, least squares mean; SEM, standard error of mean.

*Worst grade through the stuy period (24 weeeks). Grades were determined accoding to the National Cancer Institute Common Toxicity Criteria, version 4.0; †Changes in GHS/QoL and functional scales from baseline by adverse events were analysed over time until protocol termination (disease progression or 24 weeks) using linear mixed-effect model. ‡Composite categories.

Blue marks indicate statistical significance (P< .05), but not clinical meaningful. Red marks indicate both clinical significance and clinical meaningful. A difference of more than 10 points in change scores from baseline was considered clinically meaningful.
