# Supplementary material for: A prospective Phase II study to examine the relationship between quality of life and adverse events of first‐line chemotherapy plus cetuximab in patients with KRAS wild‐type unresectable metastatic colorectal cancer: QUACK trial
Source: Cancer Med. 2018 Jul 26;7(9):4217–27. doi: 10.1002/cam4.1623 (PMC6144158; doi:10.1002/cam4.1623)
Supplement: Supplementary file 4 [file CAM4-7-4217-s004.docx]

**Supplemental Information**

**Patients and methods**

***Study Design and Eligibility Criteria***

The QUACK study is a multicenter, prospective, Phase II study conducted in Japan. Detailed information with respect to the study design and patient eligibility criteria etc. has been previously described ^1^. In brief, all patients received cetuximab plus chemotherapy (FOLFOX or FOLFIRI) as a first-line treatment; the key eligibility criteria included patients aged 20 years or older with histologically confirmed unresectable mCRC, no history of prior chemotherapy (except for adjuvant chemotherapy more than 6 months prior to enrolment), an Eastern Cooperative Oncology Group Performance Status (ECOG PS) score of 0–2, wild type KRAS codons 12 and 13, at least one measurable lesion according to the Response Evaluation Criteria in Solid Tumors (RECIST) version.1.1, an estimated life expectancy of greater than three months, and adequate organ function. This study has been conducted in accordance with the Declaration of Helsinki and the Ethics Guidelines for Clinical Research by the Ministry of Health, Labor, and Welfare in Japan. Informed consent was obtained from all patients before registration. The study protocol was approved by the institutional review board or ethics committee of each participating institution and it was registered with the University Hospital Medical Information Network (UMIN) Clinical Trial Registry (UMIN000010985) on July 19, 2013.

***Treatments***

Registered patients were treated with FOLFIRI plus cetuximab or mFOLFOX6 plus cetuximab by the physicians’ discretion in each institution according to their standard clinical practice for treating mCRC. The FOLFIRI plus cetuximab regimen consisted of cetuximab (initial infusion of 400 mg/m^2^ followed thereafter by a weekly infusion of 250 mg/m2) with concurrent l-leucovorin (200 mg/m^2^) and irinotecan (150 mg/m^2^), followed by 5-fluorouracil (intravenous bolus of 400 mg/m^2^ followed by a 46 h of continuous infusion of 2,400 mg/m^2^ every 14 days). The mFOLFOX6 plus cetuximab regimen was the same as the FOLRIRI plus cetuximab regimen, but irinotecan was replaced with oxaliplatin (85 mg/m^2^). Treatment was continued until disease progression, unacceptable toxicities, death, achievement of complete response, surgical resection following conversion to operable disease, withdrawal of consent, or the physician’s decision, whichever was earlier. There were no post-study treatment limitations.

***Endpoints and Assessments***

The endpoints are the following associations: AEs and QOL, treatment efficacy and skin toxicity, and efficacy and QOL. Disease progression and the occurrence of new diseases were monitored by radiological methods (computed tomography or magnetic resonance imaging) at pre-chemotherapy (baseline) and every eight weeks during the treatment period. Treatment response was evaluated by the investigator at each institution using RECIST version 1.1. OS was defined as the time from registration until death. Progression-free survival (PFS) was defined as the time from registration to the time of progression after first-line treatment initiation or death from any cause.

Safety was assessed by monitoring AEs using physical and laboratory examinations and AE severity was graded according to National Cancer Institute’s Common Toxicity Criteria (NCI-CTC) version 4.0. An early skin reaction was defined as the worst severity of skin toxicity within eight weeks from initiation of the treatment. The survey sheets, including safety, efficacy and compliance with treatment, were collected at registration and after four, eight, 16, and 24 weeks. In addition, patient outcome was investigated two years after study initiation and one year after accrual of the last patient.

QOL was assessed at baseline and after two, four, eight, 16, and 24 weeks, and a time window of two weeks around each follow-up QOL assessment time point was accepted. If the patient did not complete the study treatment, the last QOL assessment was performed at the time of judgment of study termination or the nearest scheduled time point. The European Organisation for Research and Treatment of Cancer Quality of Life Questionnaire C30 (EORTC QLQ-C30) version 3.0 is a self-administered, cancer-specific, multidimensional questionnaire. This questionnaire was used to assess HRQOL because it is valid and reliable in the advanced cancer setting, including CRC ^2, 3^. This 30-item questionnaire contains a global health status (GHS)/QOL scale, five functional scales (physical, role, cognitive, emotional, and social), three symptom scales (fatigue, pain, and nausea/vomiting), and six single scales assessing additional symptoms (dyspnea, insomnia, appetite loss, constipation, diarrhea, and financial impact) ^2^. Scoring was completed according to the EORTC QLQ-C30 manual, and a linear transformation was used to standardize raw scores to a range between 0 and 100, with higher scores indicating better QOL on functional scales and more symptoms on symptom scales ^4^. A difference of more than 10 points in change scores from baseline was considered clinically meaningful ^5, 6^. The Dermatology Life Quality Index (DLQI), a widely validated skin-specific self-administered questionnaire ^7-9^ was used to assess skin-related QOL, and it contains 10 questions covering six domains (symptoms and feelings, daily activities, leisure, work and school, personal relationships, and treatment). The total scores range from 0 to 30, with higher scores indicating greater QOL impairment ^7^. A change in DLQI score of at least four points was considered clinically meaningful ^10^.

***Statistical Analysis***

Patients who withdrew consent before any intervention were excluded from all the analyses. QOL analyses were conducted in patients with a baseline and at least one post-baseline QOL assessment. Questionnaire compliance rates were calculated as the number of patients who completed a questionnaire at a given time point divided by the number of patients expected to be evaluable at that time point. In order to examine the impact of AEs on QOL, we analyzed the association of the worst grade of AEs with the changes in the EORTC QLQ-C30 scores from baseline throughout observation period of eight and 24 weeks. For this analysis, we used a linear mixed-effects model for repeated measures, with the intercept and slope for the study week as random effects to estimate the least squared means of the change from baseline. The impact of skin toxicity on changes in DLQI scores and the impact of treatment efficacy on changes in EORTC QLQ-C30 scores were also assessed using the same statistical analysis. The Kaplan–Meier method was used to estimate the distributions of OS, and the log-rank test was used to compare the distribution of survival time. The association between time to event endpoints and early skin toxicity was analyzed using the Cox proportional hazard model with adjustment for pre-emptive skin treatment, age, gender, ECOG PS score, chemotherapy backbone, site of primary tumor, presence of primary tumor, number of metastatic lesions, metastatic sites, and second-line chemotherapy, which calculates the adjusted hazard ratio (HR) and the 95% confidence interval (CI). For continuous variables, between-group comparisons were conducted using the Wilcoxon–Mann–Whitney test. All statistical analyses were conducted with the JMP 12 software package (SAS Institute, Cary, NC, USA).

**Conflict of interest disclosures**:

KY received speaker honoraria from Chugai, Bristol-Myers Squibb, Merck Serono, Takeda, and Eli Lilly. JS received consultant fee from Takeda Pharmaceutical Co. Ltd., and Honoraria from Tsumura Co. Ltd., and Chugai Pharmaceutical Co. Ltd. SM received honoraria from MSD. MK received honoraria from Chugai Pharmaceutical Co. Ltd., Takeda Pharmaceutical Co. Ltd., Yakult Honsha., Taiho Pharmaceutical., and Merck Serono. HH received honoraria from Chugai Pharmaceutical Co. Ltd., Yakult Honsha., Taiho Pharmaceutical., Merck Serono, and Lilly, and consultant fee from Ono Pharmaceutical, Chugai Pharmaceutical, and Merck Serono. MN received honoraria from Chugai Pharmaceutical Co. Ltd., Yakult Honsha., Taiho Pharmaceutical., Merck Serono, Lilly, Takeda Pharmaceutical Co. Ltd.,Bayer, and Otsuka. SI received honoraria from Chugai Pharmaceutical Co. Ltd., and Merck Serono. HS received honoraria from Chugai Pharmaceutical Co. Ltd., Yakult Honsha., Taiho Pharmaceutical., Merck Serono, Lilly, Takeda Pharmaceutical Co. Ltd.,and Bayer. AT received honoraria from Daiichi Sankyo, Chugai Pharmaceutical Co. Ltd., Taiho Pharmaceutical., Merck Serono, and, Takeda Pharmaceutical Co. Ltd., Bristol-Myers Squibb Japan, and speaker Bureau from Chugai Pharmaceutical Co. Ltd., Taiho Pharmaceutical., Merck Serono, and, Takeda Pharmaceutical Co. Ltd. Merck KGaA reviewed the manuscript for medical accuracy only before journal submission. The authors are fully responsible for the content of this manuscript, and the views and opinions described in the publication reflect solely those of the authors.

**Supplemental References**

1. Ooki A, Ando M, Sakamoto J, Sato A, Fujii H, Yamaguchi K. A prospective observational study to examine the relationship between quality of life and adverse events of first-line chemotherapy plus cetuximab in patients with KRAS wild-type unresectable metastatic colorectal cancer: QUACK Trial. Jpn J Clin Oncol. 2014;44: 383-387.

2. Aaronson NK, Ahmedzai S, Bergman B, et al. The European Organization for Research and Treatment of Cancer QLQ-C30: a quality-of-life instrument for use in international clinical trials in oncology. J Natl Cancer Inst. 1993;85: 365-376.

3. Byrne C, Griffin A, Blazeby J, Conroy T, Efficace F. Health-related quality of life as a valid outcome in the treatment of advanced colorectal cancer. Eur J Surg Oncol. 2007;33 Suppl 2: S95-104.

4. Fayers P, Aaronson NK, K. B. The EORTC QLQ-C30 Scoring Manual (ed 2). Brussels, Belgium, Eurpoean Organisation for Research and Treatment of Cancer. 1999.

5. Au HJ, Karapetis CS, O'Callaghan CJ, et al. Health-related quality of life in patients with advanced colorectal cancer treated with cetuximab: overall and KRAS-specific results of the NCIC CTG and AGITG CO.17 Trial. J Clin Oncol. 2009;27: 1822-1828.

6. Osoba D, Rodrigues G, Myles J, Zee B, Pater J. Interpreting the significance of changes in health-related quality-of-life scores. J Clin Oncol. 1998;16: 139-144.

7. Finlay AY, Khan GK. Dermatology Life Quality Index (DLQI)--a simple practical measure for routine clinical use. Clin Exp Dermatol. 1994;19: 210-216.

8. Lacouture ME, Mitchell EP, Piperdi B, et al. Skin toxicity evaluation protocol with panitumumab (STEPP), a phase II, open-label, randomized trial evaluating the impact of a pre-Emptive Skin treatment regimen on skin toxicities and quality of life in patients with metastatic colorectal cancer. J Clin Oncol. 2010;28: 1351-1357.

9. Peeters M, Siena S, Van Cutsem E, et al. Association of progression-free survival, overall survival, and patient-reported outcomes by skin toxicity and KRAS status in patients receiving panitumumab monotherapy. Cancer. 2009;115: 1544-1554.

10. Basra MK, Salek MS, Camilleri L, Sturkey R, Finlay AY. Determining the minimal clinically important difference and responsiveness of the Dermatology Life Quality Index (DLQI): further data. Dermatology. 2015;230: 27-33.
